# Aumento da Espessura Médio-intimal Aórtica e sua Relação com Estresse Oxidativo Elevado em Pacientes com Talassemia Menor

**DOI:** 10.36660/abc.20210666

**Published:** 2022-06-06

**Authors:** Cansu Tumer, Tayyibe Saler, Muhammed Zubeyir Aslan, Ayse Selcan Koc, Mevlüt Koc, Ozcan Erel, Salim Neselioglu, Erdinc Gulumsek, Begum Seyda Avci, Akkan Avci, Hilmi Erdem Sumbul

**Affiliations:** 1 Adana Health Practice and Research Center Adana Turquia Adana Health Practice and Research Center, Adana – Turquia; 2 Department of Medical Biochemistry University of Yıldırım Beyazıt Ankara Turquia Department of Medical Biochemistry, University of Yıldırım Beyazıt, Ankara – Turquia

**Keywords:** Talassemia Beta, Espessura Intima-Media Carotídea, Estresse Oxidativo

## Abstract

**Fundamento:**

A espessura médio-intimal (EMI) da artéria aorta abdominal (EMI-A) pode ser um marcador precoce de aterosclerose subclínica e um indicador objetivo de estresse oxidativo em pacientes com talassemia menor.

**Objetivo:**

Avaliar se as EMIs da artéria aorta e da artéria carótida (EMI-C) se alteram com estresse oxidativo, e examinar a relação entre esses parâmetros em pacientes com talassemia menor.

**Métodos:**

O estudo incluiu 80 pacientes diagnosticados com talassemia menor, e 50 indivíduos sadios com idade e sexo similares. Após procedimentos de rotina, as amostras de sangue foram coletadas dos grupos de estudo para a medida da homeostase tiol/dissulfeto e da albumina modificada pela isquemia (AMI). As medidas da EMI-C foram realizadas a partir de quatro regiões diferentes (artéria carótida externa direita e esquerda e artéria carótida interna direita e esquerda) por ultrassonografia, e a medida da EMI-A foi realizada por ultrassonografia abdominal. Um valor de p<0,05 foi definido como estatisticamente significativo.

**Resultados:**

Nos pacientes com talassemia menor, os níveis de tiol nativo e tiol total, e a razão tiol nativo/tiol total foram mais baixos, e os valores de AMI, razão dissulfeto/tiol nativo, e razão dissulfeto/tiol total foram mais altos que no grupo controle. A EMI-A foi significativamente maior no grupo de pacientes com talassemia menor que nos controles (1,46±0,37 vs 1,23±0,22 e p<0,001). Quando os parâmetros associados com EMI-A na análise univariada foram avaliados por regressão linear multivariada, EMI-A apresentou uma relação positiva, e os níveis de tiol nativo e tiol total apresentaram uma forte relação negativa com AMI (p<0,01).

**Conclusão:**

Nós demonstramos, pela primeira vez, um aumento no estresse oxidativo com a elevação da EMI-A, e valores inalterados da EMI-C em pacientes com talassemia menor.

## Introdução

A talassemia é uma doença genética que ocorre devido a uma diminuição ou ausência de uma ou mais cadeias de globinas que constituem a hemoglobina. A talassemia é herdada de maneira autossômica recessiva,^1^ em que há ocorrência de defeitos na produção de várias cadeias polipeptídicas (alfa, beta, gama ou delta), que se diferem clinicamente e bioquimicamente. A talassemia menor é uma forma mais branda da talassemia beta, com um genótipo heterozigoto e anemia level.^1^ É uma doença comum nos países do Oriente Médio e Ásia central, e países do Mediterrâneo, como a Turquia.^2^

A lesão endotelial é um componente importante do processo aterosclerótico. Em pacientes com talassemia beta, sabe-se que um aumento no acúmulo de ferro decorrente de um aumento nos processos de hemólise, transfusão e absorção intestinal, leva a uma redução na disponibilidade do óxido nítrico (NO) endotelial, e consequentemente à disfunção endotelial.^3^ Os radicais livres formados se ligam e são neutralizados por tiois; ligações dissulfeto surgem como resultado dessa reação, e se tornam tiol novamente, conduzindo a uma homeostase tiol-dissulfeto. Um desequilíbrio na homeostase causa disfunção endotelial e o início da aterosclerose.

Estudos mostraram que a medida da espessura médio-intimal da artéria carótida (EMI-C), a qual é um indicador objetivo tanto de estresse oxidativo^4 , 5^ como de aterosclerose subclínica, está aumentada em pacientes com talassemia maior.^6 - 8^ A relação entre estresse oxidativo aumentado e EMI-C aumentada é clara em muitas doenças, incluindo a beta-talassemia maior.^9 , 10^

Contudo, não existem muitos estudos avaliando EMI-C ou estresse oxidativo em pacientes com beta-talassemia menor. Somente um estudo relatou que tanto os níveis de EMI-C quanto os de estresse oxidativo estavam aumentados em um número limitado de pacientes com beta-talassemia menor.^11 , 12^ Estudos têm indicado que a medida da EMI pode ser preditiva de eventos cardiovasculares causados por aterosclerose e útil na detecção de aterosclerose subclínica.^13 - 17^ A aterosclerose é uma doença que se inicia da infância e causa o aumento da EMI da aorta abdominal (EMI-A). Muitas doenças causam um comprometimento precoce da EMI-A sem afetar a EMI-C.^18 , 19^ No presente estudo, nosso objetivo foi investigar a relação entre EMI-A, EMI-C e marcadores de estresse oxidativo, e se esses parâmetros estão alterados em pacientes com beta-talassemia menor. O estudo foi um estudo unicêntrico, caso-controle. O estudo foi aprovado pelo comitê de ética da Faculdade de Medicina da Universidade de Çukurova (13 de abril de 2018, reunião 76, decisão 88). Consentimento foi obtido dos pacientes que aceitaram em participar no estudo.

### População do estudo

Indivíduos encaminhados para o Departamento de Clínica Médica da Universidade de Ciências da Saúde e Centro de Pesquisa e Prática em Saúde, Adana, Turquia ( *Department of Internal Medicine of Adana Health Practice and Research Center / University of Health Sciences* ), entre 01 de janeiro de 2016 e 02 de março de 2018, por vários motivos, e submetidos à eletroforese de hemoglobina foram considerados elegíveis para o estudo. O estudo incluiu 80 pacientes com idade superior a 18 anos de idade, diagnosticados com talassemia menor por eletroforese de hemoglobina, que não apresentavam doença sistêmica e concederam consentimento oral e por escrito. Cinquenta indivíduos sadios, com idade e sexo similares, foram incluídos como controles. Não foram incluídos indivíduos com idade menor de 18 anos, grávidas, fumantes, etilistas, indivíduos com doenças sistêmicas (diabetes mellitus, hipertensão, insuficiência cardíaca, acidente vascular cerebral, síndrome metabólica, insuficiência renal, insuficiência hepática, câncer, doenças autoimunes), pacientes com infecções crônicas ou agudas, e aqueles que não deram consentimento verbal e escrito. Foram realizados anamnese e exame físico de todos os participantes. Foram registrados idade, sexo, altura, peso corporal, e níveis sanguíneos de nitrogênio ureico no sangue, creatinina, alanina aminotransferase, aspartato aminotransferase, proteína C reativa ultrassensível, triglicerídeos, lipoproteína de baixa densidade, hormônio tireoestimulante, e hemograma. O índice de massa corporal (IMC) foi calculado utilizando-se a fórmula padrão [peso (Kg)/ altura (m^2^). Não foram solicitados outros testes dos pacientes. Os pacientes que foram submetidos à eletroforese de hemoglobina pelo método de cromatografia líquida de alta eficiência (HPLC) e apresentaram HbA2 ≥3,5 e HbF entre 2-10% foram considerados carreadores de beta-talassemia. O hemograma foi avaliado pelo aparelho SYSMEX XE-2100i (Japão), usando o método de citometria de fluxo fluorescente. A glicemia foi medida pelo método da hexoquinase, os níveis de colesterol pelo método colorimétrico enzimático, e a creatinina medida pelo método de Jaffé, todos pelo aparelho Roche C-501 (Japão).

### Homeostase Tiol/Dissulfeto e albumina modificada pela isquemia

Para avaliação da homeostase tiol/dissulfeto, amostras de sangue foram coletadas em tubos de tampa amarela com gel, os quais foram centrifugados a 2000 rpm por 10 minutos. O soro foi separado e armazenado a -80 graus. Em seguida, as amostras foram enviadas para o Departamento de Bioquímica da Universidade de Ciências da Saúde e Centro de Pesquisa e Prática em Saúde, Adana, Turquia, e mantidas em baixa temperatura até serem analisadas pelo Prof. Dr. Özcan Erel. O índice 1 foi obtido dividindo-se dissulfeto (D) por tiol nativo (TN) (D/TN); o índice 2 foi obtido dividindo-se D por tiol total (TT) (D/TT), o índice 3 foi obtido dividindo-se TN por TT (TN/TT). As medidas foram realizadas com um analisador automático Cobas C501 (Roche-Hitachi, Mannheim, Alemanha). A albumina modificada pela isquemia (AMI) no soro foi avaliada pelo teste de ligação cobalto-albumina, aplicando-se o método espectrofotométrico. Para o teste, 50μL de cloreto de cobalto 0.1% foi adicionado a 200μL de soro do paciente e, a amostra permaneceu incubada por 10 minutos para permitir a ligação da albumina ao cobalto. Em seguida, 50μL de ditiotreitol (DTT) 1.5 mg / mL foi adicionado para medir o cobalto que não se ligou à albumina. Cobalto livre foi corado com DTT para formar um complexo colorido, e esse complexo foi medido por espectrofotometria em um comprimento de onda de 470 nm. O cobalto livre medido foi definido como o valor de AMI. Os custos dos kits foram cobertos pelo Prof. Dr. Özcan Erel, e não houve custo adicional para o nosso hospital ou para a Instituição de Segurança Social da Turquia.

### Ultrassonografia modo B das artérias carótidas e aorta abdominal ^13^

**Figura 1:**
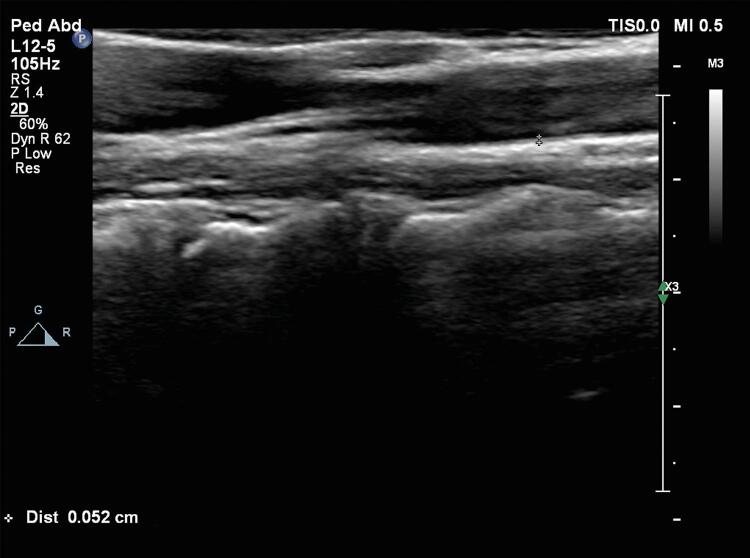
Medida da espessura médio-intimal da artéria carótida comum por ultrassonografia modo B em um paciente com talassemia menor (valor normal: 0,57 mm).

A aorta abdominal e as artérias carótidas direita e esquerda (comum e interna) foram examinadas por ultrassom com Doppler de alta resolução (Philips EPIQ 7), equipado com transdutores convexo e linear de 12 e 5 MHz de alta resolução (Philips Health Care, Bothell, WA, EUA). Todas as artérias foram estudadas tanto no corte longitudinal como no corte transversal. Todas as artérias foram escaneadas longitudinalmente para visualizar a EMI na parede arterial posterior ou distal. Todas as medidas foram realizadas nas imagens estáticas. As duas imagens de melhor qualidade foram escolhidas de cada indivíduo para análise. EMI foi definida como a distância entre a borda principal da primeira linha ecogênica e a borda principal da segunda linha ecogênica. A primeira linha representa a interface lúmen-íntima e a segunda linha representa a camada de colágeno da túnica adventícia. A EMI vascular foi medida por dois observadores independentes, cegos, utilizando-se pinças ultrassônicas. Os valores de EMI foram calculados como médias de seis medidas ( Figura 1 ).

**Figura 2:**
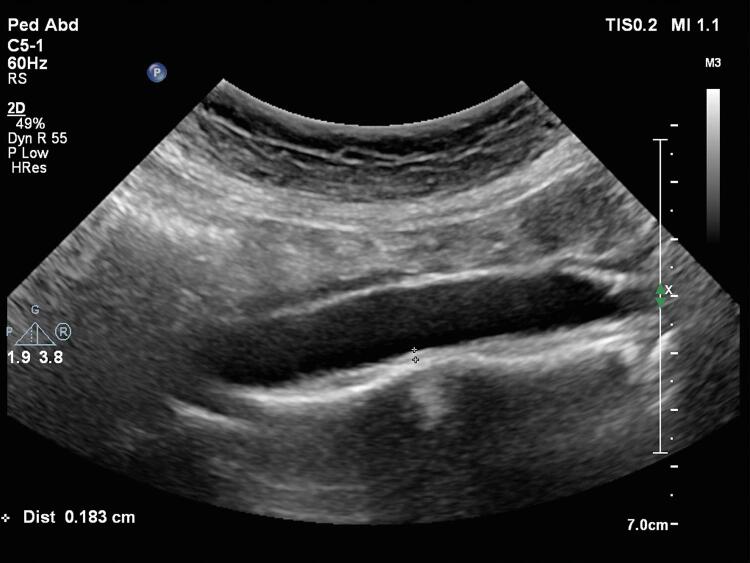
Medida da espessura médio-intimal da artéria aorta abdominal por ultrassonografia modo B mostrando valor aumentado (1,83 mm) em um paciente com talassemia menor.

Os indivíduos foram avaliados na posição supina. Para o escaneamento da artéria carótida, a cabeça dos participantes foi inclinada 45 graus para a direita. As EMIs com medidas entre 10 e 20mm (para artérias carótidas comuns) e distal (para artérias carótidas internas) da bifurcação em duas imagens de ultrassom bidimensional foram aceitas como EMI-CC e EMI-CI, respectivamente. A EMI-A foi medida da bifurcação da artéria renal à bifurcação da artéria ilíaca. A EMI medida da parede posterior da artéria abdominal foi considerada a EMI-A ( Figura 2 ).

### Análise estatística

Todas as análises foram realizadas utilizando o programa SPSS 22.0 (SPSS para Windows 22.0, Chicago, IL, EUA). Os dados categóricos foram apresentados como números e porcentagens, e comparados pelo teste do qui-quadrado. As variáveis contínuas foram expressas em média ± desvio padrão ou mediana e intervalo interquartil, conforme apropriado. A distribuição normal das variáveis contínuas foi analisada pelo teste de Shapiro-Wilk. As variáveis contínuas com distribuição normal foram comparadas pelo teste t para amostras independentes, e as variáveis que não apresentaram distribuição normal foram comparadas pelo teste de Mann-Whitney. O coeficiente kappa foi usado para avaliar a variabilidade interobservador e a variabilidade intra-observador de todas as medidas eletrocardiográficas e ecocardiográficas. A correlação de Pearson foi usada para avaliar a relação entre as variáveis contínuas. Todas as variáveis associadas à EMI-A, identificadas na análise univariada, foram em seguida avaliadas por regressão linear. Os parâmetros com distribuição normal preencheram os critérios de normalidade necessários. As variáveis significativas (p<0,01) na análise de correlação univariada foram incluídas na análise. Um valor de p<0,05 foi considerado estatisticamente significativo em todas as comparações.

## Resultados

**Figura 3:**
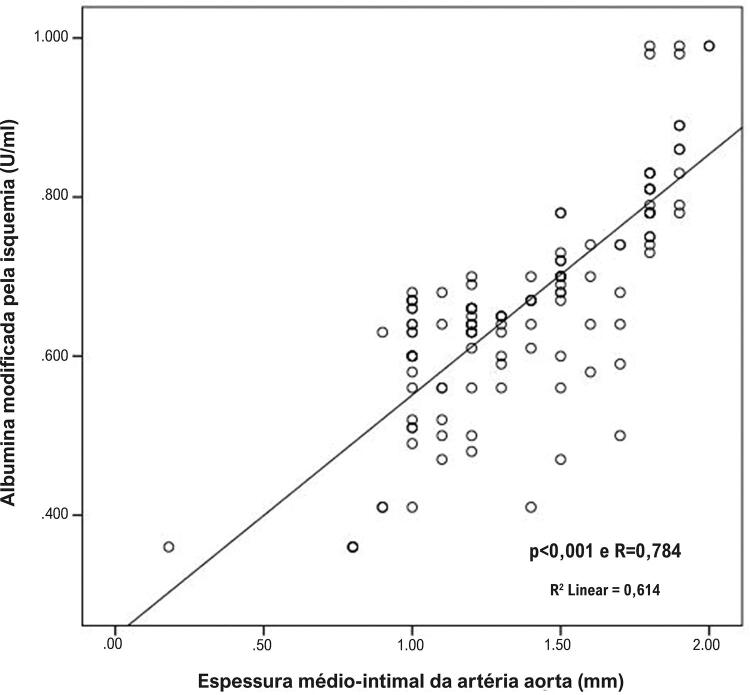
Correlação significativa entre espessura médio-intimal da artéria aorta e níveis de albumina modificada pela isquemia.

Os dados do estudo foram comparados entre pacientes com talassemia menor e controles sadios. O coeficiente kappa foi maior que 90% para todas as medidas eletrocardiográficas e ecocardiográficas – variabilidade interobservador e intra-observador para eletrocardiografia (ECG): 96% e 98% e ecocardiografia: 97% e 98%, respectivamente. As medidas de EMI foram obtidas com sucesso de todos os pacientes incluídos no estudo. Todos os dados clínicos e demográficos foram similares entre os grupos, exceto frequência cardíaca, a qual foi maior nos pacientes com talassemia menor. Todos os parâmetros bioquímicos dos dois grupos foram similares, exceto hemograma. Contagem de hemácias, hemoglobina, hematócrito, e volume corpuscular médio foram menores, e a amplitude de distribuição das hemácias foi maior nos pacientes com talassemia menor ( Tabela 1 ). Enquanto TN, TT e TN/TT foram mais baixos nos pacientes com talassemia menor, EMI, D/TN e D/TT foram mais altas que no grupo controle. Níveis séricos de D não foram diferentes entre os grupos ( Tabela 2 ). Enquanto EMI-A foi significativamente mais alto nos pacientes com talassemia menor, todos os valores de EMI-C não foram diferentes em comparação aos controles. EMI-A correlacionou-se negativamente com o nível de TT. A análise de regressão linear foi conduzida com os parâmetros que apresentaram uma relação significativa com a medida de EMI-A ( Tabela 3 ). A Tabela 4 mostra a correlação das medidas de EMI-A com os parâmetros clínicos e laboratoriais. EMI-A correlacionou-se positivamente com a pressão arterial sistólica e diastólica, níveis de TN, D e EMI, e razões D/TN e D/TT. As análises de regressão linear revelaram que a EMI-A se associou independentemente com a EMI, e níveis de TN e TT. A relação mais forte foi observada entre EMI-A e EMI ( Figura 3 ).

**Tabela 1:** Comparação dos dados demográficos e laboratoriais entre indivíduos com talassemia menor e controles sadios

	Talassemia menor n=80	Indivíduos sadios n=50	p
Idade (anos)	38,5 ± 13,9	38,4 ± 13,9	0,957
Sexo feminino n (%)	53 (%66,2)	33 (%66)	0,977
Pressão arterial sistólica (mmHg)	116 ± 6,0	117 ± 6,8	0,399
Pressão arterial diastólica (mmHg)	73,2 ± 3,6	74,3 ± 5,4	0,182
Frequência cardíaca (pulse/minute)	81,4 ± 11,3	67,3 ± 3,6	**<0,001**
Índice de massa corporal (kg/m^2^)	28,0 ± 2,5	27,4 ± 1,6	0,154
Ureia (mg/dL)	23,4 ± 5,40	23,2 ± 5,3	0,800
Creatinina (mg/dL)	0,56 ± 0,16	0,55 ± 0,16	0,867
Glicose (mg/dL)	93,5 ±9,86	92,8 ± 10,5	0,677
Aspartato aminotransferase (u/L)	19 (12,8 – 21,7)	19,5 (12,4 – 21,7)	0,961
Alanina aminotransferase (u/L)	16,6 (12,1 – 19,1)	16,6 (12,1 – 19,1)	0,940
Triglicerídeo (mg/dL)	94 (82 – 167)	102 (82 – 171)	0,858
Lipoproteína de baixa densidade (LDL) (mg/dL)	114 ± 27	115 ± 27	0,852
Hormônio tireoestimulante (uIU/dL)	1,74 ± 0,88	1,67 ± 0,92	0,679
Proteína C reativa ultrassensível (mg/dL)	0,60 (0,30 – 0,90)	0,55 (0,30 – 0,90)	0,799
Contagem de leucócitos (x10^6/μL)	7,54 ± 1,48	7,57 ± 1,51	0,903
Contagem de hemácias (x10^6/μL)	5,51 ± 0,86	4,55 ± 0,41	< 0,001
Hemoglobina (g/dL)	11,6 ± 1,45	12,8 ± 1,22	< 0,001
Hematócrito (%)	35,8 ± 4,82	38,6 ± 3,75	< 0,001
Volume corpuscular médio (fL)	65,2 ± 8,1	84,8 ± 7,1	< 0,001
Amplitude de distribuição das hemácias	16,8 ± 2,9	13,3 ± 1,5	< 0,001
Contagem de plaquetas (x10^3/μL)	249 ± 69	249 ± 71	0,990

**Tabela 2:** Comparação de parâmetros de estresse oxidativo entre pacientes com talassemia menor e indivíduos sadios (controles)

	Talassemia menor n=80	Indivíduos sadios n=50	p
Tiol nativo (μmoL)	337 ± 52	384 ± 38	<0,001
Tiol total (μmoL)	350 ± 42	417 ± 35	<0,001
Dissulfeto (μmoL)	18,2 ± 7,40	16,6 ± 3,72	0,106
Dissulfeto / Tiol nativo	6,08 ± 2,73	5,16 ± 1,23	0,010
Dissulfeto / Tiol total	5,32 ± 2,06	4,64 ± 1,21	0,020
Tiol nativo / Tiol total	89 ± 4,13	91 ± 2,25	0,026
Albumina modificada pela isquemia (unidade de absorbância)	0,70 ± 0,14	0,59 ± 0,06	<0,001

**Tabela 3:** Comparação da espessura da médio-intimal da artéria carótida e da artéria abdominal em entre pacientes com talassemia menor e indivíduos sadios (controles)

	Talassemia menor n=80	Indivíduos sadios n=50	p
EMI da artéria aorta (mm)	1,46 ± 0,37	1,23 ± 0,22	<0,001
EMI da artéria carótida comum direita (mm)	0,57 ± 0,11	0,56 ± 0,11	0,943
EMI da artéria carótida interna direita (mm)	0,56 ± 0,13	0,56 ± 0,12	0,941
EMI da artéria carótida comum esquerda (mm)	0,59 ± 0,12	0,58 ± 0,12	0,948
EMI da artéria carótida interna esquerda (mm)	0,56 ± 0,11	0,56 ± 0,10	0,940

*EMI: espessura médio-intimal.*

**Tabela 4:** Correlação entre valores de pressão arterial e parâmetros de estresse oxidativo com espessura médio-intimal em pacientes com talassemia menor

	Análise de correlação		Análise de regressão	

	p	R	p	β
Pressão arterial sistólica (mmHg)	**<0,001**	0,701	0,127	0,113
Pressão arterial diastólica (mmHg)	**<0,001**	0,720	0,127	0,089
Tiol nativo (μmoL)	**<0,001**	– 0,435	**0,002**	– 0,173
Tiol total (μmoL)	**<0,001**	– 0,721	**<0,001**	– 0,296
Dissulfeto (μmoL)	**<0,001**	0,609	0,223	0,118
Dissulfeto / Tiol nativo	**<0,001**	0,621	0,455	0,021
Dissulfeto / Tiol total	**<0,001**	0,645	0,372	0,026
Tiol nativo / Tiol total	**<0,001**	– 0,670	0,787	– 0,270
Albumina modificada pela isquemia (unidade de absorbância)	**<0,001**	0,784	**<0,001**	0,491

*R^2^_ajustado_ = 0,666.*

## Discussão

Nosso estudo apresentou novas informações à literatura sobre a talassemia menor. O primeiro e o principal resultado foi que a EMI-A, mas não a EMI-C, estava aumentada em pacientes com talassemia menor. Esse é o primeiro estudo a avaliar e a demonstrar o aumento na EMI-A nesses pacientes. Também investigamos o equilíbrio tiol-dissulfeto e os níveis de AMI para avaliar o estresse oxidativo, o qual estava aumentado nesses indivíduos. Além disso, EMI-A aumentada estava positivamente correlacionada com EMI, um dos parâmetros de estresse oxidativo, e fortemente e negativamente correlacionada com TT e TN. Embora a relação entre estresse oxidativo aumentado e EMI aumentada já é conhecida em outras doenças, esta é a primeira vez em que essa relação foi demonstrada nessa população.

O estresse oxidativo é causado por um desequilíbrio entre a produção de espécies reativas de oxigênio e o sistema antioxidante. Um dos mecanismos antioxidantes é o equilíbrio tiol/dissulfeto. A avaliação desse equilíbrio é crítica para elucidar os efeitos do estresse oxidativo sobre a patogênese das doenças e avaliar as respostas a tratamentos antioxidantes.^20^ Estudos demonstraram que um equilíbrio anormal do equilíbrio tiol/dissulfeto está envolvido na patogênese de várias doenças tais como diabetes mellitus, doenças cardiovasculares, câncer, artrite reumatoide, doença de Parkinson, doença celíaca e outras doenças inflamatórias intestinais, doença de Alzheimer e esclerose múltipla.^21 - 24^ Em nosso estudo, o equilíbrio dinâmico tiol/dissulfeto foi comparado entre indivíduos com beta-talassemia menor e indivíduos sadios controles. Ainda, avaliamos a relação entre EMI e EMI-C, já previamente avaliada em pacientes com talassemia maior. Ainda, este é o primeiro e único estudo a avaluar tanto os níveis de AMI como a homeostase tiol/dissulfeto em indivíduos com talassemia menor. Enquanto os níveis de EMI, D/TN e D/TT foram significativamente mais altas nos pacientes com talassemia menor que no grupo controle, TN, TT e TN/TT foram significativamente mais baixos. Tal fato pode ser explicado pela presença de excesso de cadeias de globina alfa livres causado pela deficiência de cadeia de globina beta, levando à formação de radicais superóxido e hidroxil e início de reações oxidativas em cadeia.^25^ Estudos epidemiológicos e ensaios clínicos mostraram que a EMI-C, determinada por ultrassonografia modo B de alta resolução, correlaciona-se positivamente com fatores de risco cardiovasculares tradicionais, e pode fornecer informação sobre risco aumentado. A ultrassonografia para avaliação de EMI-C é recomendada por diretrizes tradicionais sobre classificação de risco cardiovascular como um método de rastreamento não invasivo para aterosclerose subclínica.^14 - 17^ Estudos de autópsia mostraram que a primeira lesão aterosclerótica inicia-se na superfície dorsal da aorta abdominal distal.^26^ Embora a aorta abdominal seja uma artéria propensa à aterosclerose, a EMI-A não foi tão estudada como a EMI-C. Estudos mostraram uma correlação positiva da EMI-A com pressão arterial sistólica, frequência cardíaca, e níveis de creatinina, hormônio tireoestimulante, fator de crescimento semelhante à insulina tipo 1 e hormônio do crescimento. A investigação da aterosclerose da aorta abdominal tem o potencial de fornecer informações importantes para a avaliação do risco cardiovascular. Os aparelhos atuais de ultrassonografia e as sondas de alta resolução permitem a clara visualização da aorta abdominal e a medida da EMI-A.^18 , 19 , 27 - 29^ Foi demonstrado claramente que a medida da EMI-C está aumentada em pacientes com beta-talassemia maior.^6 - 8^ Contudo, em nosso conhecimento, a avaliação da EMI em pacientes com talassemia beta foi realizada somente em um estudo, com um número limitado de pacientes, que relatou que esses pacientes apresentaram EMI-C aumentada.^11^ A principal razão para esse fato pode ser que, em pacientes com talassemia menor, o risco de se iniciar um processo de aterosclerose subclínica em pacientes com beta-talassemia menor é menor que em pacientes com talassemia maior, e o quadro clínico atual não causaria aumento a EMI. Em nosso estudo, as medidas da C-EMI foram realizadas a partir de quatro segmentos diferentes da artéria carótida, e foi não foram encontradas diferenças nos valores de EMI entre os pacientes com talassemia menor e o grupo controle. No estudo de Gullu et al.,^11^ a medida da EMI foi realizada somente da artéria carótida comum, e o número de pacientes com talassemia menor incluídos no estudo foi a metade que em nosso estudo. Assim, nossos resultados podem ser mais significativos que os obtidos por esses autores.^11^ No entanto, para elucidar a relação entre a fisiopatologia da talassemia menor e EMI-C, mais estudos são necessários. Sabe-se que a EMI-A é um indicador mais precoce de doenças ateroscleróticas e fatores de risco para muitas doenças em comparação à EMI-C.^18 , 19 , 27 - 29^ Na literatura, não existem estudos avaliando EMI-A em pacientes com beta-talassemia. Em nosso estudo, observamos que a EMI-A foi significativamente maior nos pacientes com beta-talassemia menor que em controles sadios. Em estudos recentes sobre EMI-A como um indicador precoce de aterosclerose, um aumento na EMI-A sem aumento na EMI-C foi observado em pacientes com infarto do miocárdio, hiperparatiroidismo, e diabetes mellitus, dados que estão de acordo com os de nosso estudo.^18 , 19 , 30^

Estudos mostraram que a paraoxonase-1 e o estresse oxidativo estão elevados em pacientes com talassemia maior, contribuindo para o desenvolvimento da doença arterial coronariana e formação de placa aterosclerótica.^31^

Em outro estudo, os autores mostraram que o estresse oxidativo aumentou com a diminuição da atividade da paraoxonase-1 em pacientes com talassemia menor.^12^ Ainda, a prevalência da síndrome metabólica é relativamente alta em indivíduos com talassemia menor, o que também está de acordo com nosso estudo ao considerar a contribuição da síndrome no desenvolvimento da aterosclerose.^32^ Outro estudo também mostrou que indivíduos com talassemia menor têm um risco duas vezes maior de desenvolverem diabetes e resistência à insulina em comparação à população sem a doença.^33^

Em nosso estudo, os pacientes com talassemia menor apresentaram estresse oxidativo aumentado, com disfunção na homeostase tiol/dissulfeto e aumento na AMI; e todos esses parâmetros de estresse oxidativo apresentaram uma íntima relação com a EMI-A. Esse achado comprovou que o estresse oxidativo associou-se com EMI aumentada em pacientes com talassemia menor bem como em pacientes com talassemia maior.^10^

### Limitações

A principal limitação deste estudo foi ser um estudo unicêntrico, transversal, com um número limitado de pacientes. Outra limitação foi o fato de não havermos incluído pacientes com talassemia maior e pacientes com talassemia intermediária como grupos de estudo, uma vez que tanto a EMI-C como o estresse oxidativo estavam claramente aumentados nesses indivíduos. Se incluídos, esses parâmetros poderiam ser comparados com o grupo com talassemia menor. Outra limitação importante foi o fato de não termos realizado análise da mutação genética ou do fenótipo bioquímico proaterogênico dos pacientes com talassemia menor. A frequência de fenótipo bioquímico proaterogênico parece estar aumentada em pacientes com talassemia menor em comparação à população geral.^34^ Em nosso estudo, a análise do fenótipo bioquímico proaterogênico e da mutação genética forneceria resultados mais significativos. Ainda, a medida da EMI foi realizada por um radiologista com experiência prévia em EMI, que possui muitas publicações e tem 10 anos de experiência em ultrassonografia. No entanto, uma vez que todas as medidas foram conduzidas pelo mesmo especialista, a variabilidade interobservador não foi avaliada.

## Conclusão

Neste estudo, encontramos que a EMI-A, que pode ser avaliada de maneira não invasiva e confiável por ultrassonografia abdominal, está aumentada em pacientes com beta-talassemia menor. Além disso, os níveis de TN e TT estavam diminuídos, e os de AMI aumentados; o mecanismo antioxidante e o equilíbrio pró-oxidante/antioxidante estavam deteriorados em favor dos pró-oxidantes. Similar à relação entre o aumento no estresse oxidativo e nos valores de EMI-C, a EMI-A mostrou-se intimamente relacionada com estresse oxidativo aumentado em nosso estudo. Ainda, a avaliação da EMI-A pode ser uma ferramenta promissora na detecção de aterosclerose subclínica e na avaliação de estresse oxidativo em pacientes com beta-talassemia menor. Mais estudos com acompanhamento em longo prazo desses pacientes são necessários.
