# Supplementary material for: Aumento da Espessura Médio-intimal Aórtica e sua Relação com Estresse Oxidativo Elevado em Pacientes com Talassemia Menor
Source: Arq Bras Cardiol. 2022 Jun 6;119(3):426–35. [Article in Portuguese] doi: 10.36660/abc.20210666 (PMC9438530; doi:10.36660/abc.20210666)
Supplement: Translation

# Translation: Aortic Intima Media Thickness is Increased and Closely Related to Elevated Oxidative Stress Increases in Beta Thalassemia Minor

**Authors:** Cansu Tumer, Tayyibe Saler, Muhammed Zubeyir Aslan, Ayse Selcan Koc, Mevlüt Koc, Ozcan Erel, Salim Neselioglu, Erdinc Gulumsek, Begum Seyda Avci, Akkan Avci, Hilmi Erdem Sumbul

**Affiliations:** 1 Turkey Adana Health Practice and Research Center, Adana – Turkey; 2 Turkey Department of Medical Biochemistry, University of Yıldırım Beyazıt, Ankara – Turkey

**Keywords:** Beta-Thalassemia, Carotid Intima-Media Thickness, Oxidative Stress

## Abstract

**Background:**

Abdominal aortic intima media thickness (A-IMT) may be an early marker of subclinical atherosclerosis and an objective indicator of increased oxidative stress in beta-thalassemia minor patients.

**Objective:**

To evaluate whether aortic and carotid IMTs change with oxidative stress and to assess the relationship between these parameters in beta-thalassemia minor patients.

**Methods:**

The study included 80 patients diagnosed with beta-thalassemia minor, and 50 healthy individuals with similar age and gender. After routine procedures, blood samples were collected from the study groups for thiol-disulfide hemostasis and ischemia-modified albumin (IMA). C-IMT measurements were performed in four different regions (right and left internal and external carotid artery) by ultrasonography. In addition, A-IMT measurement was performed by abdominal ultrasonography. Statistically significant p value was set as <0.05 for all comparisons.

**Results:**

In beta-thalassemia minor patients, native thiol, total thiol and native thiol / total thiol ratio were lower, and the IMA, disulfide / native thiol ratio and disulfide / total thiol ratios were higher than in healthy control group. A-IMT measurement was significantly higher in beta-thalassemia minor group than controls (1.46±0.37 vs 1.23±0.22 and p<0.001). When the parameters associated with A-IMT in univariate analysis were evaluated by multivariate linear regression analysis, A-IMT was positively related, and native thiol and total thiol levels were negatively and closely related to IMA (p<0.01).

**Conclusion:**

We demonstrated, for the first time, that oxidative stress status increased with increased A-IMT, while C-IMT remained unchanged in beta-thalassemia minor patients.

## Introduction

Thalassemia is a genetic disease that occurs due to a decrease or absence of one or more globulin chains that make up the hemoglobin tetramer. Thalassemia is inherited in an autosomal recessive pattern.^1^ There are production defects in various polypeptide chains (alpha, beta, gamma or delta), which differ clinically and biochemically. Beta-thalassemia minor is a carrier form of beta thalassemia with heterozygous genotype and mild anemia.^1^ It is common in the Middle East and central Asia countries and Mediterranean countries like Turkey.^2^

Endothelial damage is an important part of the atherosclerotic process. In beta-thalassemia patients, it is known that an increase in iron accumulation due to increased hemolysis, transfusion and intestinal absorption, leads to a decrease in endothelial nitric oxide (NO) bioavailability, and consequently to endothelial dysfunction.^3^ The resulting oxygen radicals are bound and neutralized by thiols. Free disulfide bonds appear as a result of the reaction and turn into thiol again, leading to a thiol-disulfide homeostasis. Impaired balance causes endothelial dysfunction and atherosclerosis to begin.

It has been shown that the carotid intima media thickness (C-IMT) measurement, which is an objective indicator of both oxidative stress^4 , 5^ and subclinical atherosclerosis, is increased in patients with thalassemia major.^6 - 8^ The relationship of increased oxidative stress with increased C-IMT is clear in many diseases, including the beta-thalassemia major.^9 , 10^

However, there are not many studies in the literature evaluating C-IMT or oxidative stress in beta-thalassemia minor patients. Only one study reported that both C-IMT value and oxidative stress levels were increased in a limited number of beta-thalassemia minor patients.^11 , 12^ It has been supported that IMT measurement can be predictive of cardiovascular events caused by atherosclerosis and useful in detecting subclinical atherosclerosis.^13 - 17^ Atherosclerosis is a disease that begins in childhood and primarily increases abdominal aortic IMT (A-IMT). Many diseases have an early A-IMT involvement without affecting the C-IMT.^18 , 19^ In this study, we investigated and the relationship between A-IMT, C-IMT and oxidative stress markers, and whether these parameters are changed in beta-thalassemia minor patients.

## Methods

Our study was a single-center case-control study. The study was approved by the Ethics Committee of the Faculty of Medicine of the Cukurova University (April 13, 2018, meeting number: 76, decision number: 88). Consent of patients wishing to participate in the study was obtained.

### Study population

Individuals who were referred to the Department of Internal Medicine of Adana Health Practice and Research Center / University of Health Sciences, Adana, Turkey, between 01.01.2016 and 02.03.2018 for various reasons and who were asked for hemoglobin electrophoresis were considered eligible. The study included 80 patients older than 18 years of age, who were diagnosed with beta-thalassemia minor by hemoglobin electrophoresis, who did not have a systemic disease and gave verbal and written consent. Then, 50 healthy individuals, similar in age and gender, were included as controls. Individuals under the age of 18, pregnant women, smokers and alcohol users, those with any systemic disease (diabetes mellitus, hypertension, heart failure, cerebrovascular accident, metabolic syndrome, kidney failure, liver failure, malignancy, autoimmune diseases), patients with acute or chronic infection, and those who did not give verbal and written consent were not included in the study. Anamnesis and physical examinations of all individuals were performed. Age, gender, height, body weight, and blood levels of urea nitrogen, creatinine, alanine aminotransferase, aspartate aminotransferase, high-sensitive C-reactive protein, triglyceride, low-density lipoprotein cholesterol, thyroid stimulating hormone and complete blood count were recorded. Body mass index (BMI) was calculated using the standard formula “weight (kg) / height (m^2^)”. No additional tests were requested from the patients. Individuals who underwent hemoglobin electrophoresis with the high-performance liquid chromatography (HPLC) method and had HbA2 ≥3.5 and HbF value between 2-10% were considered as beta thalassemia carriers. Complete blood count was evaluated using the SYSMEX XE-2100i (Japan) device, by the fluorescence flow cytometry method. Glucose value was measured by the hexokinase method, cholesterol values were measured by the enzymatic colorimetric method, and creatinine values were determined by the Jaffe method, all using the Roche C-501 (Japan) device.

### Thiol-disulfide homeostasis and ischemia-modified albumin measurement

For evaluation of thiol-disulfide homeostasis, blood samples were collected into yellow top gel tubes, which were centrifuged for 10 minutes at 2000 rpm; the serum was separated and stored at -80 degrees. Later, these samples were sent to the Department of Biochemistry, Ankara Health Practice and Research Center, University of Health Sciences – and maintained in cold chain until analysis by Prof Dr Özcan Erel. Index 1 was obtained by dividing disulfide (D) by native thiol (NT) (D /NT); index 2 was obtained by dividing D by total thiol (TT) (D / TT), index 3 was obtained by dividing NT by TT (NT / TT). Measurements were made with a Cobas C501 automatic analyzer (Roche-Hitachi, Mannheim, Germany). Albumin Cobalt Binding Test was used for IMA measurement in serum and spectrophotometric measurement was performed. For this test, 50μl 0.1% cobalt chloride was added to 200μl patient serum, and the sample was incubated for 10 minutes to allow the binding of albumin with to cobalt. Then, 50μL 1.5 mg / mL dithiothreitol (DTT) was added to measure the cobalt that was not bound to albumin. Free cobalt was dyed with DTT to form a colored complex, and this complex was measured spectrophotometrically at a wavelength of 470 nm. The measured free cobalt was determined as the IMA value. The costs of the kits were covered by Prof Dr Özcan Erel, and no additional costs were incurred for our hospital or the Social Security Institution.

### B-mode ultrasonography of carotid arteries and abdominal aorta ^13^

The abdominal aorta and left and right carotid (common and internal) arteries were examined with a high-resolution ultrasound Doppler system (Philips EPIQ 7) equipped with high resolution linear (12 MHz) and convex (5 MHz) transducers (Philips Health Care, Bothell, WA, USA). All arteries were studied in both longitudinal and transversal sections. All arteries were scanned longitudinally to visualize IMT in the posterior or distal arterial wall. All measurements were made on frozen images. The two best quality images from each subject were chosen for analysis. IMT was defined as the distance from the front edge of the first echogenic line to the anterior margin of the second line. The first line represents the intima-lumen interface, and the second line represents the collagen-containing top layer of the adventitia. Vascular IMT was measured using ultrasonic calipers by two independent and blinded observers. The IMT values were defined as the average of six measurements ( Figure 1 ).

**Figure 1:** Common carotid intima-media thickness (CC-IMT) measurement by B-mode ultrasound in a patient with beta thalassemia minor (normal CC-IMT: 0.57 mm).

Subjects were examined at supine position. Patients’ head were turned 45° to the right so that the carotid artery could be scanned. IMT that measured within 10-20 mm proximal (for common carotid arteries) and distal (for internal carotid arteries) to bifurcation on two-dimensional ultrasound images were accepted as CC-IMT and IC-IMT, respectively. A-IMT was measured from the renal artery bifurcation to the iliac artery bifurcation. The IMT measured from the posterior wall of the abdominal artery was considered as the A-IMT ( Figure 2 ).

**Figure 2:** Abdominal aortic intima-media thickness (A-IMT) measurement by B-mode ultrasound showing increased A-IMT (1.83 mm) in a patient with beta thalassemia minor.

### Statistical analysis

All analyzes were performed using SPSS 22.0 (SPSS for Windows 22.0, Chicago, IL, USA). Categorical data were shown as numbers and percentages and compared with the chi-square test. Continuous variables were expressed as mean ± standard deviation or median and interquartile range, as appropriate. The normal distribution of continuous variables was analyzed by the Shapiro-Wilk test. Normally distributed continuous variables were compared with independent samples t test and variables that did not show normal distribution were compared with Mann Whitney U test. The kappa coefficient was used to evaluate the interobserver and intraobserver variability of all electrocardiographic and echocardiographic measurements. Pearson’s correlation was used to examine the relationship between continuous variables. All variables associated with A-IMT, identified in the univariate analysis, were evaluated by multivariate linear regression analysis. The normally distributed parameters met the necessary assumptions. Significant variables at a p <0.1 level in the univariate correlation analysis were included in the analysis. Statistically significant p value was set as <0.05 for all comparisons.

## Results

The study data were compared between beta thalassemia minor patients and healthy controls. Cohen kappa were above 90% for all electrocardiographic and echocardiographic measurements – inter-observer and intra-observer variability for electrocardiogram (ECG): 96% and 98%, echocardiography: 97% and 98%, respectively). IMT measurements were successfully taken from all patients included in the study. All demographic and clinical data were found to be similar between the groups, except for heart rate, that was higher in beta-thalassemia minor patients. All biochemical parameters of the two groups were similar except for blood count parameters. Red blood cell count, hemoglobin, hematocrit and mean corpuscular volume were lower in beta thalassemia minor patients, and red blood cell distribution width was higher ( Table 1 ). NT, TT and NT/TT ratio were lower in the beta-thalassemia minor patients, and IMA, and the D/NT and D/TT ratios were higher than the healthy control group, serum D level was not different between the two groups ( Table 2 ). While A-IMT value was significantly higher in beta thalassemia minor patients, all C-IMT values were not different compared to healthy controls. A-IMT negatively correlated with the TT level. Linear regression analysis was performed with parameters significantly related to A-IMT measurement ( Table 3 ). Table 4 shows the correlation of A-IMT measurements with the clinical and laboratory parameters. A-IMT positively correlated with the systolic and diastolic blood pressures, NT, D and IMA levels, and the D/NT and D/TT ratios. In linear regression analyses, A-IMT was found to be independently associated with the IMA, and NT and TT levels. The strongest relationship was found between A-IMT and IMA ( Figure 3 ).

**Table 1:** Comparison of demographic and laboratory findings between beta thalassemia minor and healthy controls

	Beta thalassemia minor n=80	Healthy control group n=50	p
Age (years)	38.5 ± 13.9	38.4 ± 13.9	0.957
Female gender n (%)	53 (%66.2)	33 (%66)	0.977
Systolic blood pressure (mmHg)	116 ± 6.0	117 ± 6.8	0.399
Diastolic blood pressure (mmHg)	73.2 ± 3.6	74.3 ± 5.4	0.182
Heart rate (pulse/minute)	81.4 ± 11.3	67.3 ± 3.6	**<0.001**
Body mass index (kg/m^2^)	28.0 ± 2.5	27.4 ± 1.6	0.154
Urea (mg/dL)	23.4 ± 5.40	23.2 ± 5.3	0.800
Creatinine (mg/dL)	0.56 ± 0.16	0.55 ± 0.16	0.867
Glucose (mg/dL)	93.5 ±9.86	92.8 ± 10.5	0.677
Aspartate aminotransferase (u/L)	19 (12.8 – 21.7)	19.5 (12.4 – 21.7)	0.961
Alanine aminotransferase (u/L)	16.6 (12.1 – 19.1)	16.6 (12.1 – 19.1)	0.940
Triglyceride (mg/dL)	94 (82 – 167)	102 (82 – 171)	0.858
Low density lipoprotein cholesterol (mg/dL)	114 ± 27	115 ± 27	0.852
Thyroid stimulating hormone (uIU/dL)	1.74 ± 0.88	1.67 ± 0.92	0.679
High sensitive C reactive protein (mg/dL)	0.60 (0.30 – 0.90)	0.55 (0.30 – 0.90)	0.799
White Blood Cell Count (x10^6/μl)	7.54 ± 1.48	7.57 ± 1.51	0.903
Red Blood Cell Count (x10^6/μl)	5.51 ± 0.86	4.55 ± 0.41	< 0.001
Hemoglobin (g/dL)	11.6 ± 1.45	12.8 ± 1.22	< 0.001
Hematocrit (%)	35.8 ± 4.82	38.6 ± 3.75	< 0.001
Mean Corpuscular Volume (fL)	65.2 ± 8.1	84.8 ± 7.1	< 0.001
Red Blood Cell Distribution Width	16.8 ± 2.9	13.3 ± 1.5	< 0.001
Thrombocyte Count (x10^3/μl)	249 ± 69	249 ± 71	0.990

**Table 2:** Comparison of oxidative stress parameters between beta thalassemia minor and healthy controls

	Beta thalassemia minor n=80	Healthy control group n=50	p
Native thiol (μmoL)	337 ± 52	384 ± 38	<0.001
Total thiol (μmoL)	350 ± 42	417 ± 35	<0.001
Disulfide (μmoL)	18.2 ± 7.40	16.6 ± 3.72	0.106
Disulfide / Native thiol ratio	6.08 ± 2.73	5.16 ± 1.23	0.010
Disulfide / total thiol ratio	5.32 ± 2.06	4.64 ± 1.21	0.020
Native thiol / total thiol ratio	89 ± 4.13	91 ± 2.25	0.026
Ischemia modified Albumin (absorbance unit)	0.70 ± 0.14	0.59 ± 0.06	<0.001

**Table 3:** Comparison of carotid and abdominal aortic intima-media thickness between beta thalassemia minor patients and healthy controls

	Talassemia menor n=80	Indivíduos sadios n=50	p
Aortic IMT(mm)	1.46 ± 0.37	1.23 ± 0.22	<0.001
Right common carotid IMT (mm)	0.57 ± 0.11	0.56 ± 0.11	0.943
Right internal carotid IMT (mm)	0.56 ± 0.13	0.56 ± 0.12	0.941
Left common carotid IMT (mm)	0.59 ± 0.12	0.58 ± 0.12	0.948
Left internal carotid IMT (mm)	0.56 ± 0.11	0.56 ± 0.10	0.940

*IMT: intima-media thickness.*

**Table 4:** Correlation between blood pressure values and oxidative stress parameters with aortic intima-media thickness (A-IMT) in patients with beta-thalassemia minor

	Correlation Analysis		Regression Analysis	

	p	R	p	β
Systolic Blood Pressure (mmHg)	**<0.001**	0.701	0.127	0.113
Diastolic Blood Pressure (mmHg)	**<0.001**	0.720	0.127	0.089
Native thiol levels (μmoL)	**<0.001**	– 0.435	**0.002**	– 0.173
Total thiol levels (μmoL)	**<0.001**	– 0.721	**<0.001**	– 0.296
Disulfide levels (μmoL)	**<0.001**	0.609	0.223	0.118
Disulfide / Native thiol ratio	**<0.001**	0.621	0.455	0.021
Disulfide / total thiol ratio	**<0.001**	0.645	0.372	0.026
Native thiol / total thiol ratio	**<0.001**	– 0.670	0.787	– 0.270
Ischemia Modified Albumin levels (absorbance unit)	**<0.001**	0.784	**<0.001**	0.491

*R^2^_adjusted_= 0.666.*

**Figure 3:** Significant correlation between aortic intima-media thickness and ischemia modified albumin levels.

## Discussion

Our study gave a lot of new information to the literature about beta thalassemia minor. The first and the main finding was that A-IMT but not C-IMT values were increased in individuals with beta-thalassemia minor. This is the first study to evaluate and to demonstrate the increase in A-IMT in these patients. We also evaluated the thiol-disulfide balance and IMA levels for oxidative stress status and showed that it was increased in beta-thalassemia minor patients. In addition, increased A-IMT was positively correlated with IMA, one of the oxidative stress parameters, and negatively and closely related to TT and NT. Although the relationship between increased oxidative stress and increased IMT is known for many diseases other than beta-thalassemia minor, this is the first time that this association was shown in this group of individuals.

Oxidative stress is caused by the unbalance between the production of reactive oxygen species and the antioxidant system. One of the antioxidant mechanisms is the thiol-disulfide balance; the evaluation of this balance is critical for elucidating the effects of oxidative stress on the pathogenesis of diseases and evaluating responses to antioxidant treatments.^20^ Studies have shown that an abnormal thiol-disulfide balance is involved in the pathogenesis of various diseases such as diabetes mellitus, cardiovascular diseases, malignancies, rheumatoid arthritis, Parkinson’s disease, celiac disease and other inflammatory bowel diseases, Alzheimer’s disease and multiple sclerosis.^21 - 24^ In our study, the dynamic thiol-disulfide balance was compared between beta-thalassemia minor individuals and healthy control group. In addition, the relationship between IMA and C-IMT, previously shown in beta-thalassemia major patients, was evaluated in beta-thalassemia minor individuals. Also, this is the first and only study to evaluate both IMA levels and thiol /D homeostasis in individuals with beta-thalassemia minor. While IMA levels, D/NT, and D/TT ratios were significantly higher in beta-thalassemia minor patients than the control group, NT and TT levels, and NT/ TT ratio were significantly lower than the control group. This may be explained by the presence of excess free alpha globin chains due to β-globin chain deficiency, leading to formation of superoxide and hydroxyl radicals and initiation of oxidative chain reactions.^25^ Epidemiological studies and clinical trials have shown that C-IMT, determined by high-resolution B-mode ultrasonography, positively correlates with traditional cardiovascular risk factors, and can provide increased risk information. Ultrasonography for C-IMT evaluation is recommended by traditional guidelines on cardiovascular risk classification as a non-invasive screening method for subclinical atherosclerosis.^14 - 17^ In autopsy studies, the first atherosclerotic lesion was shown to start from the dorsal surface of the distal abdominal aorta.^26^ Although the abdominal aorta is an artery prone to atherosclerosis, A-IMT has not been as extensively investigated as C-IMT. Studies have found a positive correlation between A-IMT and systolic blood pressure, heart rate, creatinine, thyroid stimulating hormone, insulin-like growth factor-1 and growth hormone levels. Examination of abdominal aortic atherosclerosis has the potential to provide important information for cardiovascular risk assessment. Current ultrasound devices and high-resolution probes allow clear visualization of the abdominal aorta and measurement of the A-IMT.^18 , 19 , 27 - 29^ It has been clearly shown that C-IMT measurement is increased in patients with beta-thalassemia major.^6 - 8^ However, as far as we know, IMT evaluation in beta-thalassemia minor patients was performed in a limited number of patients in only one study,^11^ which reported that this group had increased C-IMT.^11^ The most important reason for this may be that in beta-thalassemia minor patients, the risk of initiating a subclinical atherosclerotic process is lower than in beta-thalassemia major patients, and current clinical features are not at a level to increase IMT. In our study, C-IMT measurements were made from four different regions – right and left internal and external carotid artery, and it was found that IMT values were not different between beta-thalassemia minor and control groups. In the study by Gullu et al.,^11^ IMT measurement was taken from the right common carotid artery only, and the number of beta thalassemia minor patients included in the study was half as our study.^11^ Therefore, our results may be more meaningful than those of the previous report. However, to elucidate the relationship between the pathophysiology of beta-thalassemia minor and C-IMT, further studies are required. It is known that A-IMT is an earlier indicator of atherosclerotic diseases and risk factors for many diseases than C-IMT.^18 , 19 , 27 - 29^ In the literature, there is no study evaluating A-IMT in beta-thalassemia patients. In our study, A-IMT was found to be significantly greater in beta-thalassemia minor patients than in healthy controls. In recent studies on A-IMT as an early indicator of atherosclerosis, A-IMT increase without a C-IMT increase was found in patients with myocardial infarction, hyperparathyroidism, and diabetes mellitus in accordance with our study.^18 , 19 , 30^

Paraoxonase-1 and oxidative status have been shown to be increased in beta-thalassemia major patients, contributing to the development of coronary artery disease and atherosclerotic plaque formation.^31^

In another study, it was shown that oxidative stress increases with decreased paraoxonase-1 activity in beta-thalassemia minor patients.^12^ In addition, the prevalence of metabolic syndrome is relatively high in individuals with beta-thalassemia minor, which is also in accordance with our study, considering the contribution of metabolic syndrome to atherosclerosis.^32^ Another study also showed that individuals with beta-thalassemia minor are at twice the risk of diabetes and insulin resistance compared to the individuals without the disease.^33^

In our study, beta-thalassemia minor patients had increased oxidative stress, with impaired thiol-disulfide hemostasis and increased IMA; and all these oxidative stress parameters were closely related to A-IMT. This finding proved that oxidative stress was associated with increased IMT in beta-thalassemia minor patients as well as in beta-thalassemia major patients.^10^

### Limitations

The most important limitation of our study is that it was a single-center, cross-sectional study with a limited

number of patients. Another limitation is that beta thalassemia major and intermedia patients were not taken as study groups, since both C-IMT and oxidative stress were clearly increased in them. If included, these parameters could be compared with the beta thalassemia minor group. Another important limitation of our study was that we did not perform analysis of genetic mutation or of proatherogenic biochemical phenotype of patients with beta thalassemia minor. The frequency of proatherogenic biochemical phenotype has been shown to be increased in beta thalassemia minor patients compared to the general population.^34^ In our study, the analysis of the proatherogenic biochemical phenotype and genetic mutation would provide more meaningful results. Also, IMT measurement was performed by a radiologist with previous experience on IMT, who has many publications and 10 years of experience on ultrasonography. However, since all measurements were made by the same specialist, the inter-observer variability was not assessed.

## Conclusion

In the present study, we found that A-IMT, which can be evaluated non-invasively and reliably with abdominal ultrasound, was increased in patients with beta thalassemia minor. In addition, the levels of NT and TT were decreased and IMA levels were increased; the antioxidant mechanism and the prooxidant-antioxidant balance were deteriorated in favor of prooxidants. Similarly to the relationship between increased oxidative stress and elevated C-IMT reported in the literature, in our study, A-IMT was found to be closely related to increased oxidative stress. Also, the assessment of A-IMT may be a promising tool in the detection of subclinical atherosclerosis and in the evaluation of the oxidative stress status. Further studies with a long-term follow-up of beta thalassemia minor patients are warranted.
